# Fever, Cognitive Decline, and Multifocal T2 Hyperintensities on Brain MRI: A Case Report of Cytokine Release Syndrome

**DOI:** 10.7759/cureus.42274

**Published:** 2023-07-21

**Authors:** Nina M D'Amiano, Jonathan Lai, Christopher Primiani, Vivek Yedavalli, Mona N Bahouth

**Affiliations:** 1 Neurology, Johns Hopkins University School of Medicine, Baltimore, USA; 2 Radiology and Radiological Science, Johns Hopkins University School of Medicine, Baltimore, USA

**Keywords:** leukocytosis, fever, diagnostic imaging, case report, cytokine release syndrome

## Abstract

Cytokine release syndrome (CRS) is a systemic inflammatory response characterized by fever, constitutional symptoms, and multiorgan dysfunction. While most commonly associated with immunotherapy, CRS can also be incited by infections or drugs. This case details the presentation and evaluation of a 71-year-old woman with a history of primary myelofibrosis and breast cancer who presented with acute onset of altered mental status. Initial vital signs were notable for severe hypertension, tachycardia, and fever. The patient was alert and oriented only to self, with little verbal output, and spontaneously moving all extremities. The patient had a submandibular gland abscess that had been diagnosed prior to presentation via a computed tomography scan of the neck. A comprehensive analysis, including blood tests, cerebrospinal fluid (CSF) analysis, electroencephalogram (EEG), and neuroimaging, was performed. Severe leukocytosis was noted and brain MRI demonstrated scattered areas of diffusion restriction and diffuse T2 white matter hyperintensities. Serial imaging demonstrated the progression of T2 hyperintensities. Ultimately, CRS was the most likely diagnosis. In this case, the inciting event was likely an infectious etiology, suspected to be the submandibular gland abscess that was present at the time of admission. It is vital to have a high index of suspicion for CRS in patients with recent infection, drug exposure, or immune dysregulation.

## Introduction

Cytokine release syndrome (CRS) is an acute systemic inflammatory syndrome. While it is traditionally associated with immunotherapy, especially chimeric antigen receptor T-cell therapy, CRS can also be triggered by recent infection, drug exposure, or immune dysregulation [1]. Regarding pathophysiology, CRS involves increased levels of inflammatory cytokines and hyperactivation of T lymphocytes, macrophages, and endothelial cells [1]. Clinically, CRS is characterized by fever, but other symptoms are variable and depend on the organ(s) affected; associated symptoms range from mild, flu-like symptoms to severe, life-threatening manifestations of the exaggerated inflammatory response [2-6]. Similarly, laboratory findings are varied, but common laboratory abnormalities include elevated inflammatory markers, leukocytosis and/or cytopenia(s), deranged coagulation parameters, as well as elevated renal and liver function tests [1-6]. Although the diagnosis of CRS is challenging, it is important to distinguish CRS from other inflammatory disorders that present similarly but require different treatments. The treatment of CRS depends on the etiology and severity of symptoms; the cornerstone of management is supportive care, and immunosuppression is added if indicated [7]. This case details the diagnosis of a patient with CRS, which is a rare pathology and a diagnosis of exclusion.

This case report was previously presented as a poster at the 2022 Johns Hopkins Department of Medicine/Whiting School of Engineering Annual Research Retreat on September 20, 2022, the 2022 Annual Meeting of the American Neurological Association on October 23, 2022, and the 2023 Johns Hopkins Medical Student Research Symposium on February 3, 2023.

## Case presentation

A 71-year-old woman, with a history of right breast ductal carcinoma (status post lumpectomy and radiation in 2021, currently in remission) and a recent diagnosis of primary myelofibrosis, presented to the emergency department with rapid onset of confusion and altered mental status. One day prior to presentation, she reported fatigue and sleepiness. She woke up to use the bathroom and felt shaky, could not stand, and had slurred speech as noted by her husband. She originally had been scheduled for an otolaryngologic appointment for the evaluation of a submandibular gland abscess; however, due to new symptoms, she presented to the hospital. Her only outpatient medication was lisinopril for hypertension.

Upon presentation, the patient was febrile (39°C), hypertensive (203/80 mmHg), and tachycardic (heart rate of 113 bpm). She was alert and oriented only to self, with mild dysarthria and little spontaneous verbal output. She had a mild left facial weakness with a flattened nasolabial fold, her fine touch sensation was intact, and a motor exam revealed full strength apart from 4+/5 power in left knee extension, knee flexion, ankle dorsiflexion, and ankle plantarflexion.

Complete blood count showed leukocytosis of 73,500 (normal range (NR) 4.5-11K/mm^3^), hemoglobin of 7.9 (NR 12-16g/dL), and thrombocytosis of 534,000 (NR 150-350K/mm^3^). The comprehensive metabolic panel was unremarkable. The erythrocyte sedimentation rate (ESR) was elevated at 93 (NR 0-20mm/h). Cerebrospinal fluid (CSF) studies showed an elevated white blood cell (WBC) count of 1,160 (NR 0-5 mm^3^) with 905 lymphocytes, 186 neutrophils, and 70 monocytes, glucose of 28 (NR 40-70 mg/dL), protein of 401 (NR <40 mg/dL), CSF/serum glucose ratio of 0.26 (NR 0.5-0.8), and she was positive for Epstein Barr virus (EBV) by polymerase chain reaction. She had an emergent bedside incision and drainage of her submandibular abscess. Cultures from the abscess grew methicillin-susceptible Staphylococcus aureus for which piperacillin/tazobactam was initiated.

**Figure 1:**
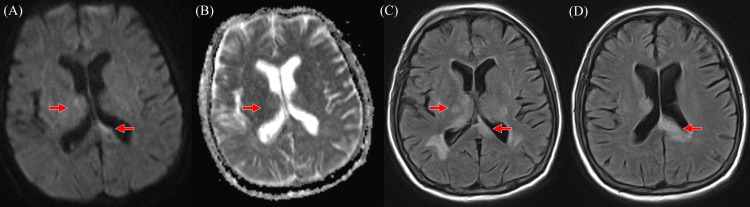
(A) Axial DWI shows subtle hyperintensity corresponding to posterior thalami and posterior periventricular white matter with inconsistent (B) ADC hypointensity and corresponding (C) axial FLAIR image at thalami and (D) splenium Images subject to motion-degradation. *Abbreviations: *ADC = Apparent Diffusion Coefficient, DWI = Diffusion-Weighted Imaging, FLAIR = Fluid-Attenuated Inversion Recovery

Magnetic resonance imaging (MRI) of the brain revealed abnormalities in the bilateral frontal lobes, left splenium, and brainstem in the form of diffusion restriction as well as multifocal T2/FLAIR white matter hyperintensities with contrast enhancement (Figure 1). Magnetic resonance angiography (MRA) showed diffuse vasculopathy, with mild stenosis of the left internal C4-C5 cavernous to clinoid carotid artery segments and mid-basilar artery. (^18^F)Fluorodeoxyglucose positron emission tomography (PET)/computed tomography scan demonstrated no abnormal uptake in the neck, chest, abdomen, or pelvis.

**Figure 2:**
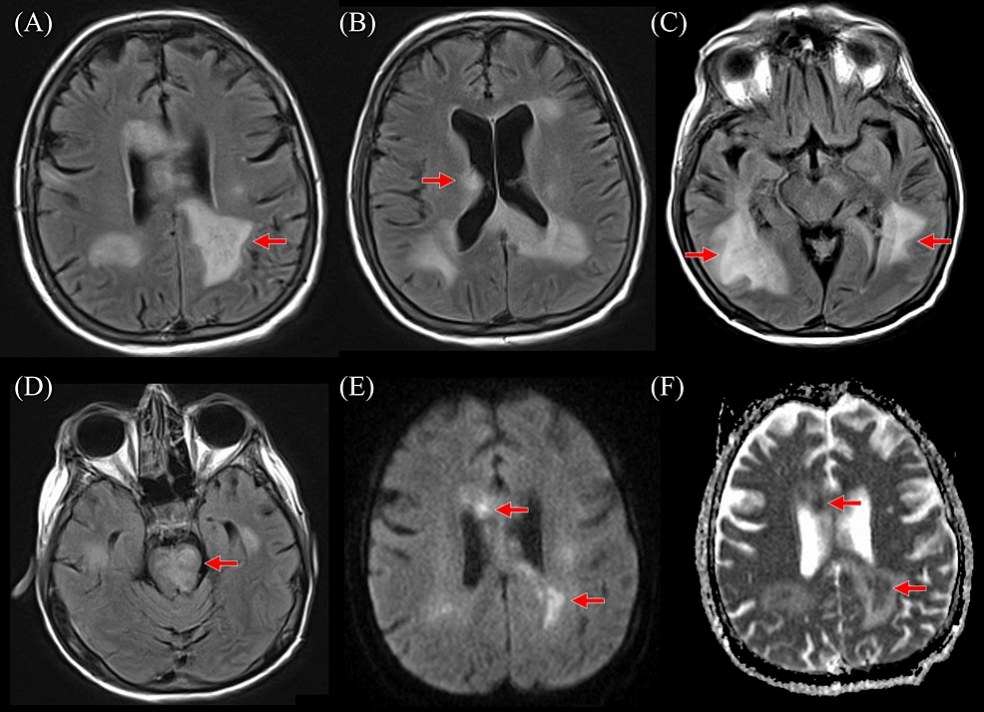
Axial FLAIR images on the first follow-up MRI at the (A) parietal white matter, (B) ganglionic, and (C) temporal lobes, and (D) pons levels demonstrate increased confluent FLAIR hyperintensities and central areas of restricted (E) diffusion with (F) ADC correlate *Abbreviations:* ADC = Apparent Diffusion Coefficient, FLAIR = Fluid-Attenuated Inversion Recovery

Over several days, the patient became uncommunicative. Repeat MRI brain showed enlargement of multiple patchy areas of T2 hyperintensity with central areas of restricted diffusion in the bilateral peritrigonal white matter, corpus callosum, midbrain/pons, and right precentral gyrus (Figure 2). Neurosurgery was consulted; however, brain biopsy was deferred due to the involvement of eloquent regions. Empiric treatment of central nervous system (CNS) lymphoma was considered based on severe neurological impairment but deferred due to risks and unclear benefits.

A third MRI brain on hospital day 24 showed improvement in T2 FLAIR hyperintensities. Later serum studies showed elevated tumor necrosis factor-alpha (TNF-α) (1.5, NR 0.56-1.40pg/mL) and interleukin-6 (IL-6) (19, NR <10 pg/mL). During her hospital stay, she developed a urinary tract infection and osteomyelitis secondary to a sacral wound.

The patient's signs and symptoms on arrival did not localize to a single anatomical location and were likely due to multifocal lesions. The differential diagnosis of altered mentation with fever, leukocytosis, and multifocal T2 FLAIR hyperintensities includes meningitis/encephalitis, CNS vasculitis, posterior reversible encephalopathy syndrome, CNS lymphoma, acute disseminated encephalomyelitis, and CRS.

The patient’s leukocytosis and positive CSF EBV were concerning for meningitis/encephalitis or CNS vasculitis incited by infection. However, the chronicity of symptoms, CSF profile, and serum and CSF cultures lacking bacterial or fungal growth made infection less likely. Furthermore, spontaneous improvement of her T2 FLAIR hyperintensities on a third brain MRI in the absence of steroid treatment was inconsistent with CNS vasculitis.

Posterior reversible encephalopathy syndrome became less likely on subsequent imaging, given the progression of areas of restricted diffusion into the bilateral temporal periventricular matter, right precentral gyrus, and posterior horn of the left lateral ventricle. This pathology typically presents with vasogenic edema involving the parieto-occipital region [8].

CNS lymphoma was deemed less likely given the acute presentation and negative PET. Spontaneous regression of her multifocal lesions further decreased suspicion for a neoplastic etiology.

Acute disseminated encephalomyelitis is an autoimmune demyelinating disease of the CNS that more commonly affects children [9]. Her age, involvement of the corpus callosum, CSF WBC >100, and spontaneous improvement without corticosteroid treatment rendered this explanation unlikely [10-11].

After consultation with neuroimmunology colleagues, the most likely diagnosis was CRS secondary to infection (likely her left submandibular gland abscess). The diagnosis of CRS was supported by her slow, spontaneous clinical improvement, down-trending ESR, and resolving leukocytosis and T2 lesions with supportive care and anti-microbial therapy. Elevated CSF WBC count and protein were nonspecific but also favored the diagnosis. CRS diagnosis was supported by elevated TNF-α and IL-6 levels as well as the development of new infections, suggesting immune dysregulation.

Over her 7.5-week hospital course, the patient was managed with anti-microbial treatment and supportive care, including delirium precautions and speech-language therapy. After six weeks in the hospital, the patient regained her ability to express basic wants, needs, and thoughts. At discharge, she was alert, oriented, and could follow simple commands. She was discharged to acute rehabilitation with plans for outpatient neurology follow-up and brain MRI. Currently, 13 months after discharge, the patient feels that she has returned to her baseline.

## Discussion

CRS is a systemic inflammatory response to drug exposure, infection, or immune dysregulation [1]. The pathophysiology involves immune cell hyperactivation and elevated levels of cytokines, causing tissue damage and associated symptoms [1]. Regarding clinical presentation, CRS is accompanied by fever, but other symptoms are variable and depend on the organ(s) affected [12]. When the CNS is involved, patients may develop neuropsychiatric findings, including aphasia, altered level of consciousness, impaired cognitive skills, and motor weakness. Neurologic symptoms usually present two to four days after the onset of CRS and may progress even after the resolution of other symptoms [6]. CRS is a clinical diagnosis, while there is no definitive test for CRS, laboratory findings may support the diagnosis, including elevated levels of c-reactive protein, ferritin, TNF-α, IL-6, IL-10, and interferon-gamma. Other supportive laboratory findings include leukocytosis, leukopenia, anemia, thrombocytopenia, and elevated D-Dimer levels [12].

Management of CRS depends on etiology and symptoms. In patients with CRS not caused by immunotherapy, the grading system and treatment algorithm are divided into four categories (Table 1) [7]. Given that our patient fell within Grade 1 and spontaneously improved clinically and on imaging, brain biopsy or corticosteroids were not indicated. Furthermore, her active infections were contraindications to immunosuppression. Fortunately, with appropriate management, even severe CRS has a relatively good prognosis [1]. A thorough understanding of the clinical presentation, differential diagnosis, and therapeutic strategies is crucial to the effective evaluation and management of CRS.

**Table 1:** CRS grading scale and treatment algorithm Source: Lee et al. [6] *Abbreviations: *FiO2 = Fraction of Inspired Oxygen; CRS: Cytokine Release Syndrome

Grade	Description	Treatment
1	Fever + constitutional symptoms.	Supportive care. Monitor fluid status. Empiric treatment for febrile neutropenia.
2	Hypotension responding to fluids. Hypoxia responding to < 40 percent FiO2.	Supportive care. Closely monitor all organ functions.
3	Hypotension managed with one pressor. Hypoxia requiring ≥ 40 percent FiO2.	Supportive care. Tocilizumab ± corticosteroids.
4	Life-threatening consequences; urgent intervention indicated.	Supportive care. Tocilizumab ± corticosteroids.

## Conclusions

In summary, the diagnosis of CRS is challenging since it can affect multiple organ systems, including the CNS. CRS should be included in the differential diagnosis for patients with multifocal neurological deficits, fever, and imaging that is non-suggestive of stroke. While CRS is traditionally associated with immunotherapy, it is critical to have a high index of suspicion for CRS in patients with recent infection, drug exposure, or immune dysregulation.
